# Effects of Genetic Background and Altitude on Sugars, Malic Acid and Ascorbic Acid in Fruits of Wild and Cultivated Apples (*Malus* sp.)

**DOI:** 10.3390/foods10122950

**Published:** 2021-11-30

**Authors:** Yajing Li, Hongxia Sun, Jindong Li, Shu Qin, Wei Yang, Xueying Ma, Xiongwu Qiao, Baoru Yang

**Affiliations:** 1 Shanxi Center for Testing of Functional Agro-Products, Shanxi Agricultural University, Taiyuan 030031, China; sakura_yj@163.com (Y.L.); anty0070@163.com (H.S.); ljdchemistry@163.com (J.L.); qinshu55@126.com (S.Q.); qiaoxiongwu@126.com (X.Q.); 2 Department of Biochemistry, Food Chemistry and Food Development, University of Turku, Turun Yliopisto, FI-20014 Turku, Finland; wei.yang@utu.fi (W.Y.); xueying.ma@utu.fi (X.M.)

**Keywords:** acids, altitude, apple, ascorbic acid, cultivars, growth site, *Malus* sp., species, sugars

## Abstract

Soluble sugars, malic acid, and ascorbic acid in 17 apple cultivars (*Malus* *domestica* Borkh.) and three wild forms (*M. pumila* ‘Saiwaihong’, *M. prunifolia* (Willd.) Borkh. and *M. micromalus* Makino) from three major apple cultivation regions in China were quantified using gas chromatography equipped with flame ionization detector (GC-FID). Fructose was the most abundant sugar, followed by sucrose, glucose, and sorbitol. Wild apples contain more sorbitol and less sucrose and were significantly more acidic than cultivated fruits. The total sugar content varied from 110 to 160 mg/g fresh fruits, total acid content from 2 to 6 mg/g, with a strong influence of genetic background and growth location. Overall, ‘Gala’, ‘Xiali’, ‘Liuyuehong’, ‘Lihong’, ‘Starking Delicious’, and ‘Starkrimson’ were characterized by higher sugar/acid ratio indicating sweeter taste compared to other cultivars. The wild apples had the highest content of ascorbic acid (0.6–0.96 mg/g). Compared to other cultivars, ‘Zhongqiuwang’, ‘Qinguan’, and ‘Nagafu No. 2′ were richer in ascorbic acid. The ascorbic acid content in the commercial cultivars was highly dependent on growth location. The content of malic acid and sucrose positively correlated to altitude, and that of glucose negatively. Malic acid positively correlated with ascorbic acid and sucrose, glucose content with ascorbic acid.

## 1. Introduction

Apple (*Malus* × *domestica* Borkh.) is one of the most widely produced and consumed fruits in the world. In 2018, the global apple production reached 86.1 million tons, with China being the largest producer (46% of global production), followed by the US (5.4%), Poland (4.6%), Turkey (4.2%), and Iran (2.9%) [1]. More than 7500 apple varieties are documented worldwide, many of which are closely related to the major adaptable genotypes, for example, ‘Red Delicious’, ‘Golden Delicious’, ‘Jonathan’ and ‘McIntosh’ [2,3,4].

As a fruit available on the market throughout the year, apple is an important part of the diet and an excellent source of nutrients and health-promoting bioactive compounds [5]. Apple components may reduce the risk of chronic diseases by various mechanisms, and their potential roles in supporting human health have been well summarized [6,7]. Research has been conducted on the phytochemical components in fruits of multiple varieties cultivated in major producing countries, such as China, Poland, Italy, New Zealand, and Austria [8,9,10,11,12]. Among the major components of apple, sugars and acids are the most important compounds, which have a strong impact on the overall organoleptic quality of the fruits [13,14]. Sweet and sour tastes are considered to be among the most important sensory properties determining the consumer preference of apples [15,16], which largely depends on the content and compositional profile of soluble sugars and organic acids, and more importantly, the balance between organic acids and sugars as reflected by sugar/acid ratio [17,18]. In mature apples, fructose is the principal sugar, followed by glucose and sucrose, while malic acid is the most dominant organic acid [12,19].

Sugars not only determine fruit sweetness, but also provide carbon sources for the synthesis of other components such as pigments, amino acids, vitamins, and aroma compounds [20]. Fruit acids have been shown to have various health-supporting activities. Malic acid has been suggested to have potential health promoting effects including regulating urinary pH, alleviating dry mouth, protecting cardiovascular system, and inhibiting α-glucosidase activity [21,22,23,24]. In addition, ascorbic acid is one of the most important nutritional factors in fruits acting as an antioxidant [25].

Many factors can influence the composition and quality of fruits [26]. Genotype is one of the key factors affecting the biochemical and physicochemical characteristics, and therefore sensory properties of the fruits of apple cultivars [18]. Both the content and profile of sugars and acids vary considerably among cultivars and species of apple [19]. The ripening time of apples is primarily determined by genetic factors but also influenced by the growth environment. Differences in fruit composition have been reported between cultivars with different ripening times during the year [27]. For example, Wu et al. found that the early-ripening apples had lower contents of soluble solids compared to the middle and late-ripening cultivars [12].

In addition to genetic factors, various pre-harvest factors, especially the temperature and light variables of the growth sites, also play an important role in determining the composition and quality of apple fruits [28]. The difference in altitudes of growth sites is associated with variation in temperature, intensity and quality of light, day/night variation, and humidity, which are known to be key factors influencing the photosynthesis and secondary metabolism of plants [28]. Therefore, the compositional characteristics and quality factors of apple fruits produced in a specific area represent the outcome of a complex interplay of genetic and environmental factors and cultivation practices applied [13,29]. The fruits of ‘Golden Delicious’ cultivated at a lower altitude (600 m) had larger fruit sizes and higher content of soluble solids and titratable acids than the fruits of the same cultivar cultivated at a higher altitude (1000 m) [28]. In another study on the composition of the fruit peel of ‘Fujiku’ apple, the high-altitude environment increased the levels of carbohydrates, anthocyanin, and some flavanol glycosides, but decreased the levels of glutamic acid and several related proteins [30].

Shanxi Province, located in the eastern part of the Loess Plateau, is one of the four major apple-producing areas in China. In 2017, the apple production of Shanxi Province was 4.45 million tons representing about 10% of the apple production in China [31]. The main apple-producing regions in Shanxi province are Yuncheng, Jinzhong, and Linfen districts, which have been included in the ‘National Plan for Advantageous Regional Development of Apples’ of China [32]. Despite the importance of global apple production, little information is available about the chemical composition of the apples produced in these regions. Thorough knowledge about compositional parameters of the apple varieties and factors influencing the composition is not only essential for the evaluation of the quality of apple fruits, but also provides important guidance for plant breeding, cultivation, and industrial processing. 

In the present study, apple fruits of seventeen cultivars and three wild forms were harvested from three major apple cultivation regions (Yuncheng City, Jinzhong City, and Linfen City), in Shanxi Province, China. In addition, fruits of two apple cultivars cultivated at high altitudes in Yunnan Province were also studied for comparison. The first aim of the study was to obtain systematic information on the overall content and composition of sugars and acids in the fruits of the major apple resources cultivated in these regions. Our aim was also to compare the composition among major cultivars and species of apples in China. Special attention was placed on the impact of growth altitudes and fruit-ripening time on the composition and content of sugars and acids in apple fruits. The results provide important information for evaluating the quality of apple fruits produced in these regions and for guiding the cultivation, plant breeding, as well as processing of apples.

## 2. Materials and Methods

### 2.1. Apple Samples

Fresh fruits of 17 apple cultivars and three wild forms were harvested from three growth regions (Yuncheng City, Jinzhong City, and Linfen City) at different altitudes, representing the major apple-producing regions in Shanxi Province, China. The altitudes of these three sites were 400–800 m, 800–1000 m, and 900–1200 m, respectively. Fruits of two cultivars ‘Qinguan’ and ‘Xinshiji’ were also collected from an orchard at a higher altitude (over 2000 m) in Yunnan Province, in addition to the three locations in Shanxi Province. Moreover, among these 20 varieties collected in the present study, 4 varieties were harvested in August 2018, 9 varieties were harvested in September, and 7 varieties were harvested in October. Table 1 presents detailed information of the samples studied including varieties, ripening times, and growth sites. The fruit samples were harvested as optimal ripe at the commercial harvest time typical of each cultivar. About 20 fruits for each cultivar and 50 fruits for each wild form were pooled from a large batch consisting of fruits collected from multiple trees, and the pooled batch was randomly divided into 5 parts, each representing one biological replicate. The fresh fruits were stored at 0 °C until analysis within 4 months after harvesting.

After the removal of seeds, the fresh apples were cut into dices and immediately mixed with dry ice and ground to a fine powder using a Knife Mill (GRINDOMIX GM 300, Retsch, Germany). The powdered samples were then stored at −20 °C until analysis.

### 2.2. Standards and Chemicals

For the identification and quantification of the soluble sugars and acids, standard compounds of fructose, sucrose, glucose, malic acid, and tartaric acid were acquired from Shanghai Yuanye Bio-Technology Co., Ltd. (Shanghai, China). Sorbitol, adonitol, and ascorbic acid were obtained from Dr. Ehrenstorfer GmbH (Augsburg, Germany).

### 2.3. Chemical Analysis

#### 2.3.1. Analysis of Sugars, Malic Acid, and Ascorbic Acid

About five grams of apple powder were weighed accurately and 15 mL MilliQ water was added. After thorough mixing and shaking at 250 rpm/min for 5 min, the mixture was centrifuged at 16,000× *g* for 10 min. The supernatant was collected and filtered into a 50 mL volumetric flask with a qualitative filtering paper. The extraction process was repeated three times, and the supernatants were combined, and the final volume was filled up to 50 mL with MilliQ water. Then, 1 mL of the extract was taken, filtered through a 0.22 μm Millipore membrane, and used for the analysis of sugars and acids, the remaining solution was used for pH determination with a pH meter (INESA, Shanghai, China). 

An aliquot of 50 μL extract was placed in auto-sampler bottles. Tartaric acid (50 μL, 0.5 mg/mL) and adonitol (50 μL, 2 mg/mL) were added as internal standards for acids and sugars, respectively. The solution was evaporated to dryness under a nitrogen flow (40 °C, 40–60 min). TMS-derivatives were prepared by adding 195 μL of Tri-Sil HTP (Thermo Fisher Scientific, Rockford, IL, USA) reagent, shaking vigorously with a Vortex (EOFO-945066, Talboys, Thorofare, NJ, USA) for 5 min, and followed by incubating at 60 °C (heat block) for 30 min. The sample was then cooled to room temperature and transferred into a 300 μL glass insert placed in an autosampler bottle for GC-FID analyses. The TMS-derivatized samples were analyzed with an Agilent 7890B gas chromatograph equipped with a flame ionization detector. The analyses were carried out with an SPB-1 column (30 m × 0.25 mm i.d. × 0.25 µm df) (Supelco, Bellefonte, PA, USA). A sample of 1 µL was injected with an auto-sampler into the injector set in split mode (split ratio of 1:10). The flow rate of the carrier gas helium was 2 mL/min. The temperature of the injector was 210 °C, and the temperature of the detector was 250 °C. The column temperature program was set at an initial temperature of 130 °C held for three 3 min, raised to 180 °C at a rate of 30 °C/min, and held at 180 °C for 10 min, raised to the final temperature of 290 °C at a rate of 40 °C/min, and held at 290 °C for 5 min.

The sugars and acids were identified by co-injection with reference compounds. The quantification of the compounds was carried out by comparing the peak areas of the analytes with those of the internal standards. Individual correction factors, determined by analysis of reference compounds, were applied to correct the difference in detector response between the internal standards and the analytes. The total content of acids and the total sugar content were calculated as the sum of individual compounds within the respective group and were used to calculate the sugar/acid ratio for each sample.

#### 2.3.2. Solid Matter Determination

For the determination of the content of soluble solid matters, juices were pressed from thawed apple flesh powders using four layers of gauze. The content of soluble solids was measured using a PR-32*α* refractometer (0–32 °Brix, Atago, Tokyo, Japan) and is expressed as °Brix.

### 2.4. Statistical Analysis

The mean values and standard deviations of each of the parameters studied including the content of individual sugars and acids as well as the total sugar content and the total content of acids, sugar/acid ratio, pH, and soluble solids were calculated. One-way analysis of variance (ANOVA) was performed for multiple comparisons, and independent-samples t-test was used to investigate the difference between the samples of the same cultivar collected at two altitudes. The above statistical analyses were all carried out with SPSS 24 (SPSS, Inc., Chicago, IL, USA). In order to show the distribution of these parameters among the samples studied, the box-plots were drawn using Origin 9 (Origin Lab Co., Northampton, MA, USA). The hierarchical cluster analysis and correlation analysis were also carried out with SPSS 24 program. Hierarchical cluster analysis was performed for the cultivated and wild apples using Squared Euclidean distance coefficient and Between-group linkage method. Correlation analysis was performed using Pearson Correlation Coefficient. Principal component analysis (PCA) was performed using The Unscrambler×10.4 (CAMO ASA, Trondheim, Norway) to identify the key factors differentiating the apple samples.

## 3. Results and Discussion

### 3.1. Compositional Profile of Sugars and Acids in Cultivated and Wild Apples

**Figure 1:**
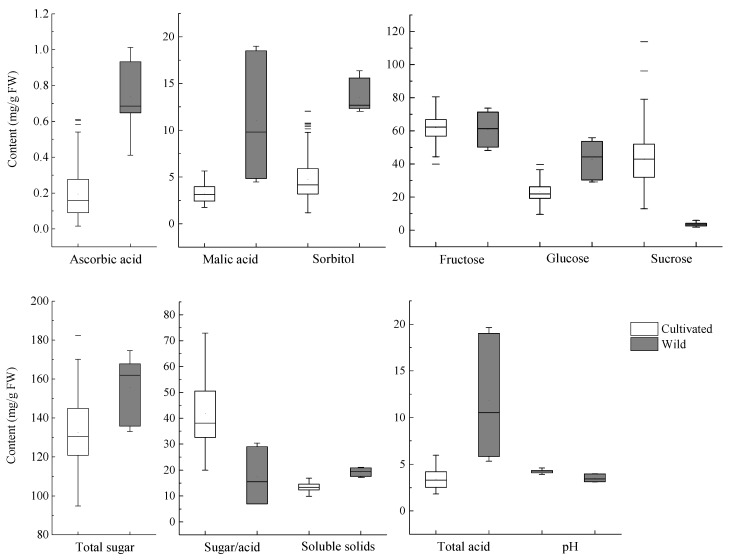
Range and distribution of sugars and acids in cultivated and wild apples. The horizontal lines in the interior of the box are the median values. The height in the box is equal to the interquartile distance, indicating the distribution for 50% of the data. Approximately 99% of samples fall inside the whiskers and the samples outside these whiskers are indicated by horizontal lines.

The sugars and acids identified and the content of these compounds in the apple samples are presented in Table 2. The major soluble sugars in the apple fruits are fructose, sucrose, and glucose; sorbitol was major sugar alcohol, which is consistent with previous reports on sugars and sugar alcohols in apple fruits of different cultivars [15,19,33]. The ranges of distribution of the content of the sugars and acids are presented in Figure 1. For most of the apple cultivars investigated in this study, fructose (46–74 mg/g fresh weight) was the most dominating sugar, followed by sucrose (17–87 mg/g), glucose (17–54 mg/g), and a sugar alcohol sorbitol (2–13 mg/g). However, there were exceptions to this order of abundance. The content of sucrose was higher than that of fructose in the fruits of the cultivar ‘Qinguan’ (sucrose, 49 mg/g; fructose, 46 mg/g) and ‘Xinshiji’ (sucrose, 87 mg/g; fructose, 57 mg/g) harvested from Yunnan province as well as the fruits of the wild apple variety *M. pumila* ‘Saiwaihong’ (sucrose, 77 mg/g; fructose, 50 mg/g) collected from Linfen, Shanxi Province. In addition, glucose was the second most abundant soluble sugar with content higher than the level of sucrose in ‘Xinshiji’ and ‘Yantai Fuji No.6′ harvested in Yuncheng and two wild apples (*M. prunifolia* (Willd.) Borkh. and *M. micromalus* Makino), although the situation was vice versa for ‘Xinshiji’ harvested in Yunnan and ‘Yantai Fuji No.6′ harvested in Linfen. Both genetic background and environment may have contributed to the variation in the relative abundance of different sugar compounds. *M. pumila* ‘Saiwaihong’ belongs to griggles with an average single fruit weight of 58.3 g, falling between the fruit sizes of cultivated and wild apples [34]. The altitude of the growth site in Yunnan Province was over 2000 m being much higher than the altitudes of the growth sites in Shanxi Province (400–1200 m), which may explain the difference in the composition compared to the fruits of the same cultivars harvested from Shanxi Province. Previous research has also shown variations in the relative abundance of sucrose and glucose in apple fruits of the same cultivars grown in different countries indicating the influence of environmental factors and horticultural practices. For example, the fruits of the cultivar ‘Golden Delicious’ had a higher content of sucrose than that of glucose in the study of Petkovsek [35] as in the current study, whereas others reported vice versa [12].

In mature fruits of 364 apple accessions originated from different countries and cultivated in the research orchard of Xingcheng Institute of Pomology of the Chinese Academy of Agricultural Sciences (Xingcheng, China), the average content of fructose, sucrose, and glucose were 36, 40, and 17 mg/g (FW), respectively. In the fruits of 321 cultivars, the average contents of fructose (35 mg/g) and sucrose (44 mg/g) were 2–3 times higher than that of glucose (14 mg/g). In contrast, the wild apples contained more fructose (41 mg/g) and glucose (37 mg/g) than sucrose (12 mg/g) [19]. The same study reported that the total sugar contents ranged from 46 to 185 mg/g (FW) in the 321 cultivars [19]. In addition, the total sugar contents in apple fruits of 8 cultivars from integrated cultivation and 11 varieties organically grown in Austria ranged 115–183 mg/g (FW) and 110–200 mg/g (FW), respectively, with fructose and sucrose being clearly more abundant than glucose. Among the 7 cultivars from integrated cultivation, ‘Jonagold’ had the highest total sugar content (183 mg/g), followed by ‘Fuji’ (140 mg/g), ‘Golden Delicious’ (135 mg/g), and ‘Gala’ (115 mg/g) [36]. In the current study, the total sugar content fell in a similar range. The lowest total sugar content was 106 mg/g f.w. in ‘Yantai Fuiji No. 6′ cultivated in Yuncheng, and the highest (169 mg/g) in ‘Saiwaihong’ from Linfen. The fruits of ‘Nagafu’ harvested from Jinzhong and Linfen had total sugar content close to 160 mg/g f.w., being the highest among the commercial cultivars. In our study, the total sugar content in ‘Gala’ (130 mg/g and 133 mg/g) cultivated in Shanxi was higher than the content reported in fruits of ‘Gala’ grown in Austria. The total sugar content in ‘Golden Delicious’ varied from 113 (Yuncheng) and 119 mg/g (Linfen) to 144 mg/g (Jinzhong).

Among the organic acids, malic acid is known to be the most abundant, accounting for about 90% of the total organic acids in ripe apple fruits [19]. Consistent with previous studies, malic acid is the dominant organic acid in the fruits of apple cultivars and species investigated in this study. The content of malic acid ranged 2–19 mg/g FW, the highest content in the two wild forms (9.7 in *M. micromalus* Makino and 18.7 in *M. prunifolia* (Willd.) Borkh.) (Table 2 and Figure 1). Among the commercial cultivars studied, ‘Golden Delicious’ harvested from Linfen had the highest content of malic acid (5 mg/g f.w.), whereas the lowest levels (around 2 mg/g f.w.) were found in ‘Liuyuehong’, ‘Starkrimson’, ‘Starking Delicious’, and ‘Xiali’ cultivated in Jinzhong, and ‘Gala’ cultivated in Yuncheng.

In a study of 18 commercial cultivars, the content of total acids ranged from 6.3–17.9 mg/g (FW) [36]. Ma et.al [19] quantitatively analyzed the malic acid in mature fruits of 364 apple accessions, and the content ranged from 0.5 to 22.7 mg/g (FW), the levels being significantly higher in wild apples than in commercial apple cultivars. High content of malic acid is known to contribute to sourness, bitterness, and astringency of fruits and berries. Therefore, it can be anticipated that wild apples are generally characterized by a higher intensity of sour taste and astringent mouthfeel.

The sugar/acid ratio ranged from 7 in the fruits of the wild apple species *M. micromalus* Makino to 68 in those of ‘Liuyuehong’ cultivated in Jinzhong. The sugar/acid ratio is an important compositional parameter with a strong impact on the sensory properties of fruits. A high sugar/acid ratio is directly correlated to high intensities of sweetness. Previous research showed that apple fruits with sugar/acid ratios below 20 have a strong sour taste and are suitable only for processing, while fruits with sugar/acid ratios higher than this value are often perceived as sweet and proper for direct consumption as fresh fruits [13]. Although all the commercial cultivars investigated in the current study had sugar/acid ratios above 20, there was a wide range of variation among the samples of different cultivars and different growth locations, indicating possible differences in sensory properties of the fruits. In contrast to the low sugar/acid in the wild forms *M. micromalus* Makino and *M. prunifolia* (Willd.) *Borkh.*, *M. pumila* ‘Saiwaihong’ has sugar/acid ratio close to 30. Indeed, ‘Saiwaihong’ has been cultivated as a commercial variety due to its good sensory properties. 

Ascorbic acid is an important antioxidant in apples. The ascorbic acid content in most of the cultivated apples ranged from 0.1 to 0.4 mg/g f.w., whereas the levels in three wild forms were higher (0.6–1 mg/g, respectively). Among commercial cultivars, the highest ascorbic acid content was found in ‘Qiufu No. 1′ cultivated in Yuncheng (0.45 mg/g f.w.) as well as ‘Yantai Fuji No. 3′ and ‘Zhongqiuwang’ harvested from Linfen (0.37 mg/g f.w.). Kumar et al. [37] reported ascorbic acid content to be 0.22 and 0.29 mg/g in the fresh fruits of ’Golden Delicious’ grown at altitudes of 1400 and 1800 m, respectively, which is close to the levels found in the fruits of the same cultivar grown in Jinzhong (800–1000 m, 0.26 mg/g) and Linfen (900–1200 m, 0.28 mg/g) in our present study. It is noteworthy that ‘Saiwaihong’ has the highest content of ascorbic acid (0.96 mg/g) among all the samples studied. A previous study reported ascorbic acid contents between 0.029 and 0.256 mg/g FW in whole fruits of 30 ancient cultivars of Belgian apples [38]. Ascorbic acid mainly is located in the peel of apples [39]. Bassi and colleagues reported that the content of ascorbic acid in pulp varied in the range of 0.001–0.139 mg/g (FW) in 64 different apple cultivars, while the content in the peel was 0.049–0.521 mg/g (FW) [40]. It is noteworthy that the ascorbic acid contents in whole wild apples of ‘Saiwaihong’ were higher than the contents reported in the pulp and peel of 64 commercial apple cultivars. All figures and tables should be cited in the main text as Figure 1, Table 1, etc.

### 3.2. Comparison between Commercial Cultivars and Wild Apples

In this study, we investigated the content of sugars and acids in 17 cultivated and 3 wild species (Table 2 and Figure 1). In general, fructose was the most abundant among sugar in cultivated and wild apples, followed by sucrose and glucose in cultivated apples whilst followed by glucose and sucrose in wild apples. A previous study showed that the average concentrations of sucrose were 2–3 times higher than that of glucose in mature fruits of 321 apple cultivars, while the average contents of glucose were over 3 times higher than that of sucrose in 43 forms of wild apples [19]. This is likely the result of continuous selection since the sweetness of sucrose is higher than that of glucose. In our study, the sorbitol contents in the fruits of three wild forms were over 12 mg/g, being much higher than the levels in the commercial cultivars (*p* < 0.01). Moreover, the three wild forms had higher soluble solid matters (above 17%) in the juice, compared with the commercially cultivated apples (average 13.5%, *p* < 0.01).

In addition, cultivated apples had lower content of ascorbic acid and malic acid, higher pH values, and higher sugar/acid ratios, compared to wild apples (*p* < 0.01). It has been shown that Asian consumers prefer very sweet apples with low intensity of acidic flavor [41]. The total acid content showed a significant difference between wild apple species and the commercially cultivated varieties (*p* < 0.01), whereas no significant difference in the total concentration of sugars was observed between *M. micromalus* Makino and cultivated apples. Thus, low content of acids has likely been a key criterion for selection during apple breeding. Malic acid is the major contributor to the acidity of apples. High sugar/acid ratio and low content of malic acid are often directly associated with high intensities of the sweetness of apple fruits [18]. The fruits of the three wild apple species contained more malic acid (9.7 mg/g, 18.7 mg/g, and 4.78 mg/g fresh weight, respectively) than the commercial cultivars (average 3.19 mg/g, *p* < 0.01). This was in agreement with a previous study, where wild apples were reported to have an average content of malic acid over 2.2 times higher than the levels found in the cultivated apples [19]. *M. pumila* ‘Saiwaihong’ contained malic acid at a level of close to 5 mg/g, which is within the range of the content determined in commercially cultivated apples. It is worth noting that *M. pumila* ‘Saiwaihong’ is a wild apple in the traditional sense, but it has been approved as a variety in 2018 for commercial cultivation in China. The sugar/acid ratios in the wild apples were significantly lower than the values of the cultivated apples. Especially, the sugar/acid ratio of two wild types (*M.* *prunifolia* (Willd.) Borkh. and *M. micromalus* Makino) were 15.71 and 6.98, respectively.

It has been reported that cultivated apples are characterized by larger fruit size and higher content of sugars when compared to the wild forms [35,42]. Nevertheless, in our study, no clear differences were found in the total sugar content between the commercial cultivars and the wild forms. Therefore, our study confirmed Ma’s finding, suggesting that fruit acidity, instead of sweetness, has undergone selection during apple domestication [19]. The higher soluble solids of the wild apples compared to the commercial cultivars in this study were mostly due to their higher contents of malic acid and ascorbic acid, not because of differences in sugar contents. 

As expected, the aqueous extracts of the fruits of the wild species had lower pH values (below 4) due to the higher total content of acids compared with the cultivated apples.

**Figure 2:**
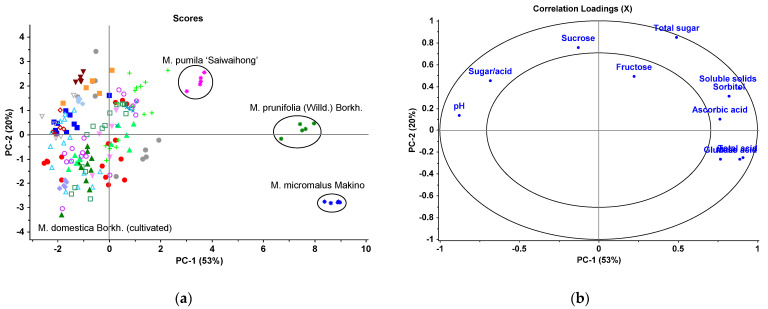
PCA model of cultivated and wild apples: (**a**) Scores plot; (**b**) Loadings plot.

To further demonstrate the difference between the commercially cultivated and wild apples, a principal component analysis (PCA) model was constructed (Figure 2). The first two principal components (PCs) explained 73% of the variance of the data. In the scores plot PCA (Figure 2a), the three wild species were clearly separated from the cultivated apples. The closer the samples are located on the plot, the more similar the compositional characteristics of the samples. In the PCA loadings plot (Figure 2b), the PC1 (53%) represented the major components of acids (malic acid, total acid, ascorbic acid), some sugars (glucose and sorbitol), and soluble solids on the positive side, pH and sugar/acid on the negative side. The three wild species had similar characteristics highly explained by PC1 by relatively high contents of ascorbic acid, total acids, glucose, sorbitol, and soluble solids, but low values of pH and sugar/acid ratio among the samples investigated. In contrast, the cultivated apples located on the negative side of PC1 show the opposite characteristics. The PC2, explaining 20% of the variance, represented the content of sucrose and the total sugar content. The PC2 separated the three wild forms based on their sugar content, especially the content of sucrose in the order: *M. pumila* ‘Saiwaihong’ > *M.* *prunifolia* (Willd.) Borkh. > *M. micromalus* Makino.

### 3.3. Comparison among Commercial Cultivars within Each Growth Region

**Figure 3:**
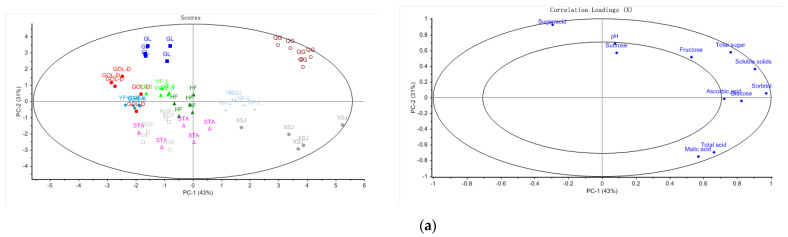

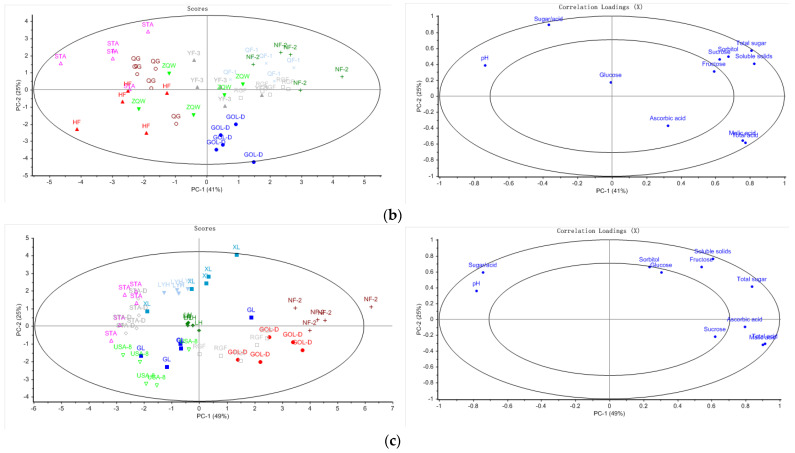
PCA models and loading plots of cultivars within each growth region. (**a**) Yuncheng. (**b**) Linfen. (**c**) Jinzhong.

Due to the impact of the growth environment on fruit quality, it is important to compare the fruit composition among cultivars grown in the same regions. Separate PCA models were constructed to demonstrate the compositional difference among different cultivars grown in the same region (Figure 3). Among the cultivars grown in Yuncheng (Figure 3a), ‘Qinguan’ was characterized by high content of total sugars, fructose, sorbitol, ascorbic acid, and soluble solids, whereas ‘Xinshiji’ stood out due to the high content of malic acid and ascorbic acid. ‘Gala’ clearly differed from other cultivars by high sugar/acid ratio. The lowest sugar/acid ratios were found in the fruits of the cultivars ‘Xinshiji’ (27.6), ‘Starkrimson’ (28.1), and ‘Red General Fuji’ (32.0) (Table 2). Detailed statistical comparisons in each of the compositional parameters among the cultivars grown in Yuncheng are presented in Table S1.

Among the cultivars grown in Linfen (Figure 3b), ‘Naga Fu No. 2’ had higher content of sugars (fructose, sucrose, and total sugars) and soluble solids, whereas ‘Golden Delicious’ had high content of malic acid, ascorbic acid, and total acids. However, ‘Starkrimson’ was clearly separated from other cultivars by high sugar/acid ratio. This was clearly in contrast with the situation in Yuncheng. Detailed statistical comparisons in each of the compositional parameters among the cultivars grown in Linfen are presented in Table S2.

Within Jinzhong region (Figure 3c), ‘Liuyuehong’ and ‘Xiali’ were characterized by a higher sugar/acid ratio compared to other cultivars. ‘Starkrimson’ and ‘Starking Delicious’ were also associated with high sugar/acid ratios. Similar to the situation in Linfen, ‘Naga Fu No. 2’ had higher content of sugars (fructose, sucrose, and total sugars) and soluble solids, whereas ‘Golden Delicious’ had high content of malic acid, ascorbic acid, and low sugar/acid ratio. Detailed statistical comparisons in each of the compositional parameters among the cultivars grown in Jinzhong are presented in Table S3.

### 3.4. Hierarchical Cluster Analysis of Cultivated Apples Based on Sugars and Acids Subsection

**Figure 4:**
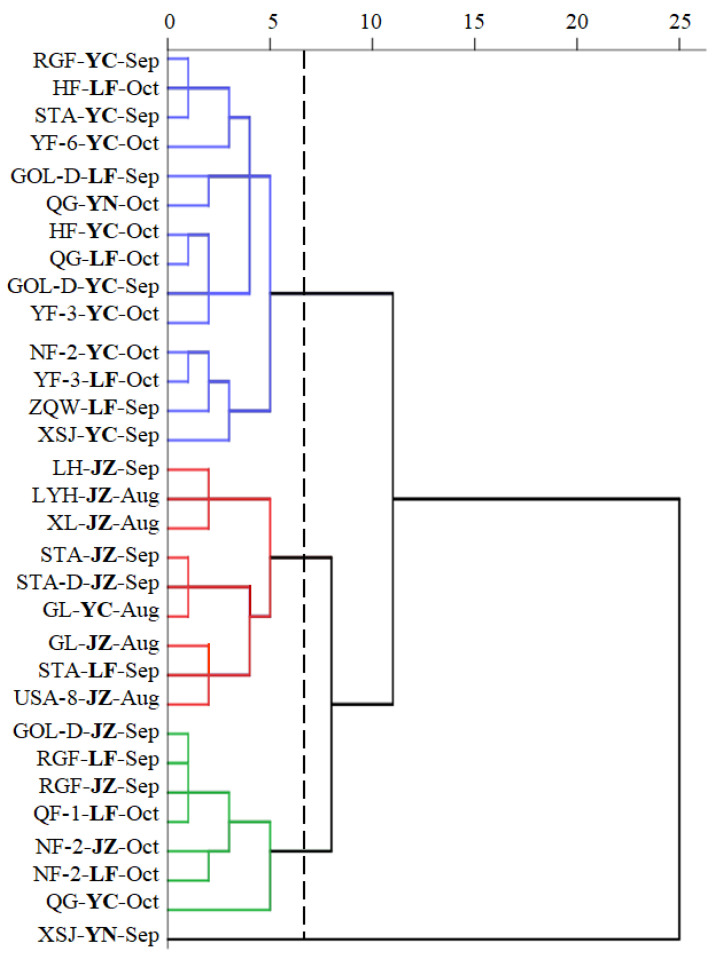
Hierarchical clustering analysis of sugars and acids of the 17 cultivated apples (A total of 31 apple samples studied). Serial nomenclature consists of the abbreviation of the cultivated apples, growth sites and ripening times. The abbreviation of cultivated apples is in Table 1. YC is Yuncheng City, Shanxi Province, JZ is Jinzhong City, Shanxi Province, LF is Linfen City, Shanxi Province and YN is Yunnan Province. Aug is August, Sep is September and Oct is October. Blue lines refer to Cluster 1, Red lines refer to Cluster 2, Green lines refer to Cluster 3, and the black line from ‘XSJ-YN-Sep’ refers to Cluster 4.

In view of the differences in sugars and acids in cultivated apples, we performed a hierarchical cluster analysis to identify 17 cultivated apples (a total of 31 samples), taking into account all the variables (Figure 4). The optimum number of clusters identified for analysis was 4, which was grouped by the elbow method. Cluster 1 (blue lines) included 14 samples, cluster 2 (red lines) included 9 samples, cluster 3 (green lines) included 7 samples, while ‘Xinshiji’ collected from Yunnan Province was clustered into a separate group (cluster 4).

Within each cluster, cultivars with similar profiles of sugars and acids were grouped together into smaller subgroups. For example, within cluster 2, ‘Lihong’, ‘Liuyuehong’, ‘Xiali’ formed a subgroup characterized by high sugar contents and high sugar/acid ratios; the second and the third subgroups consisted of ‘Starkrimson’ and ‘Gala’ cultivated in Yuncheng and Jinzhong, as well as ‘Starking Delicious’ and ‘USA-8′. It is important to notice that the cultivated apples of the same cultivars were grouped into different clusters or sub-groups, depending on the growth sites. Especially, the samples of ‘Starkrimson’, ‘Golden Delicious’, ‘Red General Fuji’ and ‘Nagafu No. 2′ collected from three growth sites and the samples of ‘Xinshiji’ and ‘Qinguan’ collected from two growth sites were separated into two clusters, which underlined the impacts of environmental conditions on the apple quality. The impact of ripening time and growth site will be discussed in the following sections.

Furthermore, it is interesting to note that all the apple samples of the early-ripening cultivars harvested in August were clustered into cluster 2, whereas the samples ripening and harvested in October were clustered into cluster 1 and cluster 3. All clusters included samples ripened and harvested in September. The possible link between the ripening time and the content of the sugars and acids in apple fruits was investigated using Principal Component Analysis (PCA) and Pearson correlation coefficients, which are presented and discussed in Section 3.4.

### 3.5. Association of Content of Sugars and Acids with Ripening Time of Cultivated Apples

**Figure 5:**
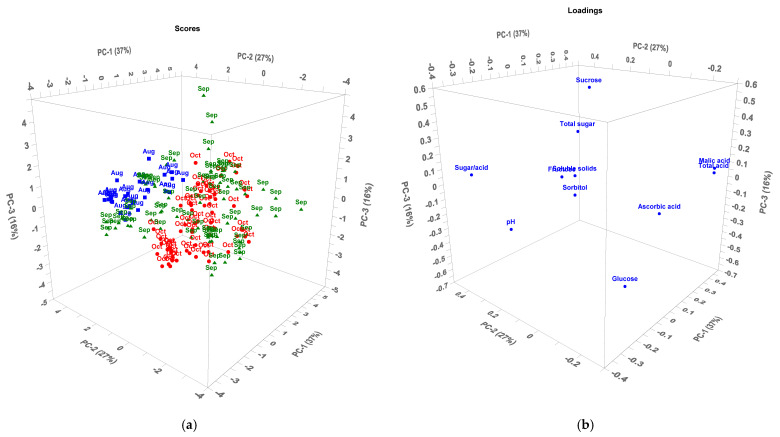
PCA model of cultivated apples by ripening time: (**a**) Scores plot; (**b**) Loadings plot. Square with Aug logo in blue font represents samples collected at August, Positive triangle with Sep logo in green font represents samples collected at September, and Dot with Oct logo in red font represents samples collected at October.

Figure 5 shows the PCA plot for the 17 cultivars harvested in August, September, and October, representing early, medium, and late-ripening groups, respectively. The first three principal components (PCs) explained 80% of the variance of the data. In the PCA scores plot, apple samples harvested in August and apple samples harvested in October were clearly divided into two groups. The samples harvested in August were distributed on the left, which were characterized as relatively high values of pH and sugar/acid as well as higher content of fructose. In contrast, the samples harvested in October located on the right and were characterized by higher contents of acids (ascorbic acid, malic acid and total acid) and glucose. The samples harvested in September were scattered across the PCA plot overlapping with the samples of the previous two groups.

For further understanding, the Pearson correlation coefficients were applied to study the correlation between the composition of apples and the ripening time (Table 3). Late ripening time was positively correlated with total acid (r = 0.358, *p* < 0.01), malic acid (r = 0.345, *p* < 0.01), ascorbic acid (r = 0.273, *p* < 0.01) and glucose (r = 0.544, *p* < 0.01), but negatively correlated with fructose (r = −0.199, *p* < 0.05), sucrose (r = −0.199, *p* < 0.05) and sugar/acid (r = −0.503, *p* < 0.01). 

The results of PCA analysis and Pearson correlation coefficients indicated that the apple samples of early-ripening cultivars harvested in August had higher sugar content, especially sucrose and fructose, whereas the samples of the late-ripening cultivars harvested in October had a higher content of acid and glucose.

### 3.6. Effect of Latitude of Growth Site on Sugars and Acids of Cultivated Apples

**Figure 6:**
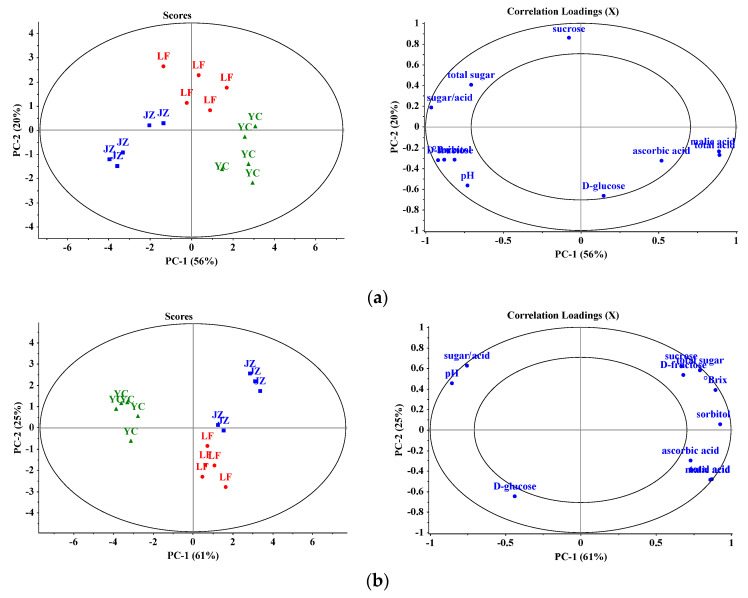

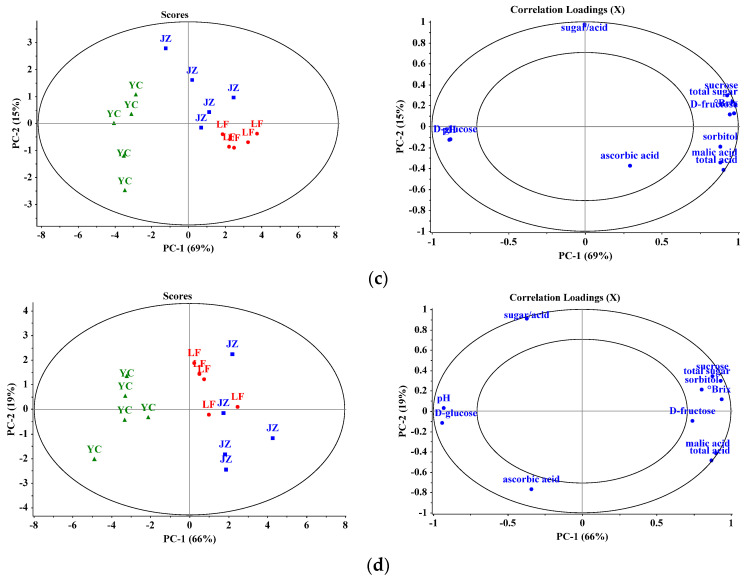
PCA model of cultivated apples in Yuncheng City (YC), Jinzhong City (JZ) and Linfen City (LF). (**a**) ‘Starkrimson’. (**b**) ‘Golden Delicious’. (**c**) ‘Red General Fuji’. (**d**) ‘Nagafu No. 2′.

PCA plots were constructed to further investigate the effect of growth sites on apple composition of each of the four cultivars, ‘Starkrimson’, ‘Golden Delicious’, ‘Red General Fuji’ and ‘Nagafu No. 2′ collected from three growth locations at different altitudes: Yuncheng (low altitude 400–800 m), Jinzhong (middle altitude 800–1000 m), and Linfen (high altitude 900–1200 m). In the PCA scores plot, the samples were clearly grouped according to the growth sites, and the samples from Yuncheng were further separated from the samples from the other two growth regions. As shown in PCA and the loadings plots, for the three cultivars ‘Golden Delicious’ (Figure 6a), ‘Red General Fuji’ (Figure 6b), and ‘Nagafu No. 2′ (Figure 6c), the samples from Yucheng (low altitude) were characterized by higher content of glucose and higher values of pH and sugar/acid ratio, but lower contents of malic acid and ascorbic acid, in comparison with the samples of the same cultivars harvested from Jinzhong (middle altitude) and Linfen (high altitude). For ‘Starkrimson’, the sample from Yuncheng was richer in malic acid and ascorbic acid than the samples from the two other locations (Figure 6d). For all the varieties, the samples from Yuncheng were clearly characterized by a lower level of sucrose and total content of sugars compared to the samples from Jinzhong and Linfen. 

These three growth areas are similar in longitude and latitude but differed clearly in altitude. Thus, besides genetic factors, environmental factors such as altitude likely have affected the content of soluble sugars, malic acid, and ascorbic acid in the apples. The samples of Jinzhong and Linfen showed somehow similar composition likely due to the similar altitudes of these two areas. Previous studies have shown that there are altitude-associated differences in the quality of apples, and apples from lower altitude are generally described as being sweeter than the samples from higher altitude, which tend to be sourer [28]. In a recent study, the malic acid content in fruits of ‘Golden Delicious’ (6.5 vs. 4.9 mg/g) and ‘Red Delicious’ (7.1 vs. 4.4 mg/g) was higher in fruits grown at an altitude of 1800 m compared with the fruits harvested from lower altitude (1400 m) [37].

The correlation between altitude and composition of apple cultivars was further analyzed through Pearson correlation coefficients (Table 3). The results indicated that altitude was positively correlated with malic acid (r = 0.342, *p* < 0.01), sucrose (r = 0.517, *p* < 0.01), total content of acids (r = 0.331, *p* < 0.01), and total content of sugar (r = 0.284, *p* < 0.01) but negatively correlated with glucose (r = −0.444, *p* < 0.01), sugar/acid (r = −0.218, *p* < 0.01), and pH values (r = −0.312, *p* < 0.01). 

Therefore, according to the findings of the PCA and Pearson correlation analysis, as the altitude increases, the content of malic acid and sucrose increases while the content of glucose decreases. In Table 2, the detailed statistical comparison among the different altitudes of not only these four cultivars but also other cultivars collected at more than one site also indicated the above conclusions. For most of the cultivars, the contents of total acid and total sugar showed a positive correlation with altitude, while sugar/acid and pH were correlated negatively with altitude, which was also coincident with the results of the Pearson correlation analysis.

Previous research has shown that latitude has an impact on carbohydrate accumulation in apple peels. At high altitude (750 m) the content of free sugars increased in the peel of apples, while the levels of amino acids decreased compared to low altitude (20 m) [30].

### 3.7. Correlations between Components of Cultivated Apples 

The Pearson correlation coefficients between sugars and acids of the commercially cultivated apples are shown in Table 4. 

Correlations were found between individual sugars and acids in the apple samples. Sorbitol was positively correlated with other individual sugars and acids. Sorbitol was first formed as the product of photosynthesis in leaves and was then converted to fructose, glucose, or malic acid [15,43]. Malic acid content was positively correlated with ascorbic acid content (r = 0.443, *p* < 0.001) and sucrose content (r = 0.252, *p* = 0.002). Previously, the content of malic acid was found to be negatively correlated with sucrose content in the fruits of apple cultivars [19]. In agreement with previous findings [19], our present study showed that the content of glucose was positively correlated with ascorbic acid content (r = 0.316, *p* < 0.001), and negatively correlated with sucrose content (r = −0.572, *p* < 0.001) likely due to the conversion of sucrose to glucose and fructose [44]. In contrast to the results of our study and the study of Ma et al. [27], the research of Celik and colleagues showed a positive correlation among fructose, glucose, and sucrose content in some ancient apple cultivars [45]. 

As expected, the content of soluble solids was positively correlated with the content of all the sugars and acids, since all these components in apples contributed to °BRIX values. As expected, the pH value correlated negatively with the acid content and the content of individual acids but positively with the sugar/acid ratio. Total sugar was positively correlated with all the acids and sugars except glucose. In addition, the ratio of sugar/acid was negatively correlated with total acid content (r = −0.866, *p* < 0.001) but not significantly correlated with total sugar content. Previous studies have shown that the total acid content varies to a larger extent among cultivars than the sugar content in apple fruits [46,47]. This indicates total acid content to be the major contributing factor to variation in flavors, especially sweetness and sourness, among different apple cultivars. Therefore, more attention should be paid to the acid content in plant breeding and fruit processing.

Bioactive molecules are really important parts of the chemical composition of apples. Previously we reported the phenolic compounds in the same cultivars and wild forms of apples included in this study [48]. A total of 10 major phenolic compounds were quantified, and the influences of altitude and genetic background on these compounds were studied. The results indicated that the three wild apples have different dominant phenolic compounds, and the commercial cultivar ‘Qinguan’ was different from other cultivars in its high chlorogenic acid content but low proanthocyanidin content. The phenolic compounds also differed among apple cultivars based on geographical growth conditions and cultivation practice. For example, the contents of procyanidin B2 and (−)-epicatechin showed a negative correlation with altitude, while other phenolic compounds were all correlated positively with altitude [48]. Our findings of sugars, acids, and phenolic compounds of 20 cultivars and forms of apples grown in different geographical environments provide important information for the selection of materials for breeding and processing, as well as guidance for suitable location and methods for targeted cultivation. 

## 4. Conclusions

The content and composition of sugars and acids are important for the quality of apple fruits. Compared with cultivated apples, the fruits of the wild apple species contained significantly higher levels of acids (malic acid), lower levels of sucrose as well as lower sugar/acid ratio. The commercial cultivars varied largely in the content of sugars, malic acid, ascorbic acid, and sugar/acid ratio. ‘Gala’, ‘Xiali’, ‘Liuyuehong’, ‘Lihong’, ‘Starking Delicious’, and ‘Starkrimson’ were characterized by higher sugar/acid ratio indicating sweeter taste compared to other cultivars. The ascorbic acid content in the commercial cultivars was highly dependent on growth location. Compared to other cultivars, ‘Zhongqiuwang’, ‘Qinguan’, and ‘Nagafu No. 2’ were richer in ascorbic acid regardless of the growth location. In addition to genetic factors, growth sites also affected the compositions of apples. The results from this study demonstrated that malic acid, sucrose, and total sugar content positively correlated with altitude, but glucose and the sugar/acid ratio correlated negatively. Malic acid is the major organic acid in apple fruits. High content of malic acid may increase the taste of acidity, bitterness, and astringency, therefore negatively contributing to the sensory properties of apple fruits. In this study, malic acid content was positively correlated with ascorbic acid content and sucrose content. Glucose content was positively correlated with ascorbic acid content and negatively correlated with sucrose content. Further, early-ripening cultivars tended to have higher sugar content and sugar/acid ratio and lower acid content than late-ripening cultivars. The current research provides important guidance for the breeding, selection, cultivation, and processing of apples.

## Figures and Tables

**Table 1:** Information of apple samples studied.

Apples	Abbreviation	Ripening Time	Growth Site ^1^	Altitude (m)
**Cultivated Apples (*Malus domestica* Borkh.)**				
Gala	GL	August	Yuncheng	400–800
			Jinzhong	800–1000
Xiali	XL	August	Jinzhong	800–1000
American No. 8	USA-8	August	Jinzhong	800–1000
Liuyuehong	LYH	August	Jinzhong	800–1000
Lihong	LH	September	Jinzhong	800–1000
Starking Delicious	STA-D	September	Jinzhong	800–1000
Zhongqiuwang	ZQW	September	Linfen	900–1200
Xinshiji	XSJ	September	Yuncheng	400–800
			Yunnan	Over 2000
Starkrimson	STA	September	Yuncheng	400–800
			Jinzhong	800–1000
			Linfen	900–1200
Golden Delicious	GOL-D	September	Yuncheng	400–800
			Jinzhong	800–1000
			Linfen	900–1200
Red General Fuji	RGF	September	Yuncheng	400–800
			Jinzhong	800–1000
			Linfen	900–1200
Nagafu No. 2	NF-2	October	Yuncheng	400–800
			Jinzhong	800–1000
			Linfen	900–1200
Huimin Fuji	HF	October	Yuncheng	400–800
			Linfen	900–1200
Yantai Fuji No. 3	YF-3	October	Yuncheng	400–800
			Linfen	900–1200
Yantai Fuji No. 6	YF-6	October	Yuncheng	500–800
Qiufu No. 1	QF-1	October	Linfen	900–1200
Qinguan	QG	October	Yuncheng	400–800
			Linfen	900–1200
			Yunnan	Over 2000
**Wild Apples (*Malus* genus)**				
*M. pumila* ‘Saiwaihong’		September	Linfen	900–1200
*M. prunifolia* (Willd.) Borkh.		September	Jinzhong	800–1000
*M. micromalus* Makino		October	Jinzhong	800–1000

^1^ Yuncheng, Jinzhong, and Linfen represent three growth sites in Shanxi Province, and Yunnan represents the growth site in Yunnan Province.

**Table 2:** Content of individual sugars and acids in different apple samples (mg/g fresh weight without core). Each mean is the average of 5 replicates. Different letters following the values of the same cultivars indicate a statistically significant difference between the samples harvested from different altitudes. The different letters in the comparison between cultivated and wild apples indicate statistically significant differences.

Cultivars	Growth Sites	Malic Acid (mg/g)	Ascorbic Acid (mg/g)	Fructose (mg/g)	Sorbitol (mg/g)	Glucose (mg/g)	Sucrose (mg/g)	Total Acid (mg/g)	Total Sugar (mg/g)	Sugar/Acid	pH	Soluble Solids (°Brix)
**Cultivated Apples (*Malus domestica* Borkh.) Collected from One Site**												
Xiali	Jinzhong	2.12 ± 0.25	0.11 ± 0.02	74.23 ± 5.12	5.77 ± 0.71	22.37 ± 2.90	43.19 ± 3.57	2.23 ± 0.26	145.56 ± 8.31	65.73 ± 5.21	4.18 ± 0.03	15.60 ± 1.01
American No. 8	Jinzhong	2.39 ± 0.33	0.13 ± 0.03	58.81 ± 2.97	4.66 ± 0.93	10.39 ± 0.82	53.91 ± 6.60	2.52 ± 0.35	127.77 ± 8.94	51.46 ± 7.97	4.30 ± 0.07	12.44 ± 0.60
Liuyuehong	Jinzhong	1.95 ± 0.03	0.20 ± 0.04	63.99 ± 0.85	10.52 ± 0.25	18.04 ± 0.30	53.48 ± 1.63	2.15 ± 0.05	146.03 ± 2.83	68.04 ± 0.96	4.55 ± 0.01	14.64 ± 0.11
Lihong	Jinzhong	2.49 ± 0.02	0.11 ± 0.05	65.68 ± 0.61	4.79 ± 0.26	18.77 ± 0.09	54.12 ± 0.37	2.60 ± 0.06	143.35 ± 1.04	55.14 ± 1.52	4.22 ± 0.01	13.98 ± 0.04
Starking Delicious	Jinzhong	2.10 ± 0.14	0.05 ± 0.03	67.52 ± 2.24	4.01 ± 0.51	20.18 ± 1.91	34.05 ± 2.92	2.14 ± 0.12	125.76 ± 4.26	58.92 ± 5.34	4.44 ± 0.05	13.30 ± 0.31
Zhongqiuwang	Linfen	3.22 ± 0.33	0.37 ± 0.04	69.43 ± 2.00	2.99 ± 0.38	20.56 ± 2.16	34.61 ± 12.73	3.59 ± 0.37	127.59 ± 13.23	35.73 ± 4.15	4.03 ± 0.07	13.74 ± 0.81
Yantai Fuji No. 6	Yuncheng	2.37 ± 0.08	0.07 ± 0.02	60.27 ± 0.70	2.38 ± 0.04	27.57 ± 0.24	15.81 ± 3.02	2.44 ± 0.10	106.03 ± 2.78	43.42 ± 0.75	4.29 ± 0.01	11.76 ± 0.09
Qiufu No. 1	Linfen	4.07 ± 0.31	0.16 ± 0.07	70.81 ± 3.66	6.61 ± 1.03	25.24 ± 1.31	44.90 ± 3.86	4.24 ± 0.30	147.57 ± 4.83	34.93 ± 2.12	4.09 ± 0.03	15.48 ± 0.52
**Comparison among Different Sites of the Same Commercial Cultivars (*Malus domestica* Borkh.)**												
Gala	Yuncheng	2.11 ± 0.14 ^b^	0.08 ± 0.02 ^a^	66.58 ± 1.55 ^a^	2.78 ± 0.45 ^a^	21.40 ± 0.62 ^a^	41.15 ± 4.40 ^b^	2.19 ± 0.15 ^b^	129.92 ± 5.63 ^a^	59.43 ± 2.44 ^a^	4.35 ± 0.08 ^a^	13.14 ± 0.26 ^a^
	Jinzhong	2.81 ± 0.38 ^a^	0.10 ± 0.06 ^a^	64.85 ± 5.33 ^a^	2.98 ± 0.74 ^a^	18.05 ± 2.09 ^b^	48.47 ± 2.74 ^a^	2.90 ± 0.41 ^a^	132.85 ± 9.75 ^a^	46.16 ± 4.04 ^b^	4.17 ± 0.07 ^b^	13.36 ± 0.99 ^a^
Xinshiji	Yuncheng	4.39 ± 0.57 ^a^	0.25 ± 0.05 ^a^	62.97 ± 3.64 ^a^	8.70 ± 2.08 ^a^	36.43 ± 1.92 ^a^	22.51 ± 7.78 ^b^	4.64 ± 0.56 ^a^	127.44 ± 8.80 ^b^	27.63 ± 1.87 ^b^	4.20 ± 0.10 ^a^	14.88 ± 0.75 ^a^
	Yunnan	3.80 ± 0.59 ^a^	0.18 ± 0.03 ^b^	56.59 ± 4.11 ^b^	3.27 ± 0.48 ^b^	16.65 ± 2.60 ^b^	87.22 ± 17.68 ^a^	3.98 ± 0.61 ^a^	164.70 ± 13.00 ^a^	41.93 ± 4.91 ^a^	4.24 ± 0.07 ^a^	13.84 ± 0.30 ^b^
Starkrimson	Yuncheng	3.75 ± 0.29 ^a^	0.24 ± 0.23 ^a^	53.17 ± 3.27 ^b^	3.30 ± 0.56 ^b^	23.49 ± 2.46 ^a^	31.41 ± 4.85 ^b^	3.99 ± 0.34 ^a^	111.37 ± 5.70 ^b^	28.07 ± 2.25 ^c^	4.33 ± 0.05 ^b^	12.00 ± 0.35 ^b^
	Jinzhong	2.05 ± 0.06 ^c^	0.05 ± 0.02 ^a^	65.1 ± 4.20 ^a^	5.03 ± 1.17 ^a^	21.89 ± 1.78 ^a^	33.11 ± 3.49 ^b^	2.10 ± 0.08 ^c^	125.13 ± 5.68 ^a^	59.73 ± 4.44 ^a^	4.57 ± 0.04 ^a^	13.62 ± 0.72 ^a^
	Linfen	2.61 ± 0.35 ^b^	0.09 ± 0.03 ^a^	54.98 ± 2.79 ^b^	3.31 ± 0.87 ^b^	21.68 ± 1.25 ^a^	46.63 ± 8.84 ^a^	2.71 ± 0.35 ^b^	126.59 ± 10.91 ^a^	47.31 ± 6.29 ^a^	4.24 ± 0.05 ^c^	12.06 ± 0.70 ^b^
Golden Delicious	Yuncheng	2.49 ± 0.30 ^c^	0.10 ± 0.04 ^b^	52.75 ± 4.27 ^b^	1.60 ± 0.27 ^b^	22.31 ± 1.22 ^a^	38.83 ± 6.64 ^b^	2.59 ± 0.33 ^c^	113.36 ± 4.65 ^b^	44.32 ± 5.48 ^a^	4.41 ± 0.06 ^a^	11.78 ± 0.11 ^c^
	Jinzhong	4.52 ± 0.20 ^b^	0.26 ± 0.12 ^a^	66.91 ± 2.85 ^a^	4.31 ± 0.78 ^a^	19.65 ± 1.66 ^b^	53.79 ± 8.36 ^a^	4.78 ± 0.21 ^b^	143.50 ± 10.02 ^a^	30.10 ± 2.68 ^b^	4.08 ± 0.04 ^b^	14.14 ± 0.63 ^a^
	Linfen	5.05 ± 0.27 ^a^	0.28 ± 0.11 ^a^	54.06 ± 3.07 ^b^	3.54 ± 0.52 ^a^	22.49 ± 2.39 ^a^	40.58 ± 5.57 ^b^	5.32 ± 0.36 ^a^	118.65 ± 5.91 ^b^	22.39 ± 2.17 ^c^	4.01 ± 0.05 ^c^	12.70 ± 0.47 ^b^
Red General Fuji	Yuncheng	3.21 ± 0.21 ^c^	0.18 ± 0.14 ^a^	56.70 ± 2.10 ^c^	2.81 ± 0.41 ^b^	25.00 ± 1.23 ^a^	26.18 ± 4.93 ^b^	3.39 ± 0.23 ^c^	107.74 ± 7.05 ^b^	31.97 ± 4.03 ^b^	4.17 ± 0.02 ^a^	11.58 ± 0.38 ^c^
	Jinzhong	3.68 ± 0.42 ^b^	0.20 ± 0.04 ^a^	65.5 ± 2.63 ^b^	3.64 ± 1.11 ^b^	20.33 ± 0.84 ^b^	52.37 ± 4.50 ^a^	3.88 ± 0.44 ^b^	140.58 ± 7.40 ^a^	36.49 ± 3.08 ^a^	4.06 ± 0.07 ^b^	13.40 ± 0.54 ^b^
	Linfen	4.23 ± 0.30 ^a^	0.30 ± 0.19 ^a^	69.12 ± 2.98 ^a^	5.30 ± 0.80 ^a^	19.01 ± 1.81 ^b^	53.53 ± 3.62 ^a^	4.53 ± 0.16 ^a^	145.77 ± 4.64 ^a^	32.17 ± 0.71 ^b^	4.00 ± 0.06 ^b^	14.00 ± 0.34 ^a^
Nagafu No. 2	Yuncheng	3.26 ± 0.23 ^c^	0.33 ± 0.08 ^a^	61.11 ± 1.06 ^b^	5.77 ± 1.04 ^b^	32.30 ± 1.83 ^a^	31.92 ± 3.62 ^b^	3.60 ± 0.25 ^c^	131.10 ± 2.66 ^b^	36.56 ± 2.07 ^a^	4.31 ± 0.02 ^a^	13.46 ± 0.59 ^b^
	Jinzhong	5.01 ± 0.60 ^a^	0.28 ± 0.09 ^a^	74.04 ± 2.02 ^a^	7.68 ± 1.23 ^a^	21.42 ± 2.09 ^b^	55.71 ± 9.48 ^a^	5.29 ± 0.67 ^a^	157.81 ± 10.23 ^a^	30.32 ± 5.10 ^a^	4.01 ± 0.02 ^b^	15.48 ± 0.75 ^a^
	Linfen	4.25 ± 0.48 ^b^	0.25 ± 0.05 ^a^	64.37 ± 3.46 ^b^	7.53 ± 1.07 ^a^	23.64 ± 2.52 ^b^	63.04 ± 7.18 ^a^	4.50 ± 0.51 ^b^	158.00 ± 6.64 ^a^	35.36 ± 3.27 ^a^	4.04 ± 0.09 ^b^	15.06 ± 0.47 ^a^
Huimin Fuji	Yuncheng	3.03 ± 0.17 ^a^	0.19 ± 0.07 ^a^	55.46 ± 3.93 ^a^	3.78 ± 0.32 ^a^	28.49 ± 3.11 ^a^	35.53 ± 7.46 ^a^	3.22 ± 0.20 ^a^	120.93 ± 5.68 ^a^	37.67 ± 2.81 ^a^	4.30 ± 0.02 ^a^	12.80 ± 0.34 ^a^
	Linfen	3.34 ± 0.32 ^a^	0.18 ± 0.09 ^a^	57.44 ± 3.58 ^a^	2.87 ± 0.74 ^b^	23.28 ± 1.97 ^b^	27.25 ± 9.40 ^a^	3.52 ± 0.37 ^a^	110.84 ± 11.07 ^a^	31.75 ± 4.70 ^b^	4.16 ± 0.06 ^b^	11.60 ± 1.08 ^b^
Yantai Fuji No. 3	Yuncheng	2.39 ± 0.07 ^b^	0.09 ± 0.04 ^b^	57.44 ± 0.75 ^b^	3.27 ± 0.13 ^b^	30.82 ± 0.56 ^a^	28.46 ± 2.82 ^b^	2.47 ± 0.11 ^b^	119.98 ± 3.74 ^b^	48.53 ± 1.22 ^a^	4.24 ± 0.02 ^a^	12.82 ± 0.08 ^b^
	Linfen	3.59 ± 0.47 ^a^	0.37 ± 0.09 ^a^	65.34 ± 2.49 ^a^	3.99 ± 0.47 ^a^	29.11 ± 2.15 ^a^	37.65 ± 6.69 ^a^	3.96 ± 0.53 ^a^	136.08 ± 5.68 ^a^	34.86 ± 5.04 ^b^	4.07 ± 0.06 ^b^	14.76 ± 0.34 ^a^
Qinguan	Yuncheng	2.70 ± 0.08 ^b^	0.45 ± 0.10 ^a^	65.78 ± 1.59 ^a^	8.83 ± 0.51 ^a^	35.50 ± 0.67 ^a^	41.01 ± 7.28 ^a^	3.15 ± 0.17 ^b^	149.12 ± 5.97 ^a^	47.47 ± 4.34 ^a^	4.48 ± 0.03 ^a^	15.86 ± 0.13 ^a^
	Linfen	3.30 ± 0.25 ^a^	0.26 ± 0.19 ^b^	51.84 ± 0.61 ^b^	5.47 ± 0.85 ^b^	23.39 ± 2.34 ^b^	43.43 ± 6.82 ^a^	3.56 ± 0.41 ^ab^	122.29 ± 6.08 ^b^	34.84 ± 4.91 ^b^	4.30 ± 0.08 ^b^	12.34 ± 0.15 ^b^
	Yunnan	3.64 ± 0.42 ^a^	0.12 ± 0.04 ^b^	46.24 ± 4.40 ^b^	4.16 ± 0.41 ^c^	19.36 ± 1.16 ^c^	48.93 ± 7.77 ^a^	3.76 ± 0.43 ^a^	117.2 ± 12.15 ^b^	31.72 ± 6.10 ^b^	4.44 ± 0.03 ^a^	11.52 ± 0.57 ^c^
**Comparison between Commercial Cultivars and Crabapples (*Malus* genus)**												
*M. pumila* ‘Saiwaihong’	Linfen	4.78 ± 0.19 ^c^	0.96 ± 0.06 ^a^	49.62 ± 0.91 ^c^	12.24 ± 0.14 ^c^	30.04 ± 0.61 ^c^	76.88 ± 3.16 ^a^	5.74 ± 0.23 ^c^	168.78 ± 4.65 ^a^	29.44 ± 0.78 ^b^	3.98 ± 0.01 ^b^	17.46 ± 0.17 ^c^
*M. prunifolia* (Willd.) Borkh.	Jinzhong	9.72 ± 0.31 ^b^	0.65 ± 0.15 ^b^	71.81 ± 1.55 ^a^	15.70 ± 0.43 ^a^	54.11 ± 1.81 ^a^	21.07 ± 2.37 ^c^	10.36 ± 0.45 ^b^	162.7 ± 4.86 ^a^	15.71 ± 0.37 ^c^	3.42 ± 0.01 ^c^	20.92 ± 0.19 ^a^
*M. micromalus* Makino	Jinzhong	18.73 ± 0.28 ^a^	0.60 ± 0.11 ^b^	61.25 ± 1.01 ^b^	12.63 ± 0.14 ^b^	44.11 ± 0.98 ^b^	17.00 ± 0.97 ^c^	19.33 ± 0.34 ^a^	134.98 ± 1.27 ^b^	6.98 ± 0.09 ^d^	3.13 ± 0.00 ^d^	19.38 ± 0.08 ^b^
*M. domestica* Borkh. (cultivated) *	Yuncheng Jinzhong Linfen	3.19 ± 0.95 ^d^	0.20 ± 0.13 ^c^	62.63 ± 6.79 ^b^	4.77 ± 2.27 ^d^	23.54 ± 5.69 ^d^	40.71 ± 12.98 ^b^	3.39 ± 1.02 ^d^	131.65 ± 15.93 ^b^	42.14 ± 12.60 ^a^	4.23 ± 0.17 ^a^	13.51 ± 1.38 ^d^

The letters a, b, c, and d marked the significant statistical differences (*p* < 0.05) within each comparison. * Values of contents in *M. domestica* Borkh. (cultivated) was the average of all the cultivated apples.

**Table 3:** Pearson’s correlation coefficients between altitude and ripening time and 11 indicators of apple cultivars.

		Malic Acid	Ascorbic Acid	Fructose	Sorbitol	Glucose	Sucrose	Total Acid	Total Sugar	Sugar /Acid	pH	Soluble Solids
Ripening time	r	0.345 **	0.273 **	−0.199 *	0.057	0.544 **	−0.199 *	0.358 **	−0.074	−0.503 **	−0.128	−0.014
	*p*	<0.001	<0.001	0.013	0.480	<0.001	0.013	<0.001	0.361	<0.001	0.111	0.860
Altitude	r	0.342 **	0.082	−0.071	−0.014	−0.444 **	0.517 **	0.331 **	0.284 **	−0.218 **	−0.312 **	0.051
	*p*	<0.001	0.308	0.379	0.867	<0.001	<0.001	<0.001	<0.001	0.006	<0.001	0.532

*: *p* ≤ 0.05; **: *p* ≤ 0.01.

**Table 4:** Pearson’s correlation coefficients between 11 indicators of apple cultivars.

		Ascorbic Acid	Fructose	Sorbitol	Glucose	Sucrose	Total Acid	Total Sugar	Sugar /Acid	pH	Soluble Solids
Malic acid	r	0.443 **	−0.009	0.160 *	0.076	0.252 **	0.993 **	0.273 **	−0.862 **	−0.686 **	0.206 *
	*p*	<0.001	0.915	0.046	0.346	0.002	<0.001	0.001	<0.001	<0.001	0.010
Ascorbic acid	r		0.127	0.295 **	0.316 **	0.039	0.545 **	0.240 **	−0.458 **	−0.285 **	0.335 **
	*p*		0.114	<0.001	<0.001	0.626	<0.001	0.003	<0.001	<0.001	<0.001
Fructose	r			0.402 **	0.032	0.085	0.009	0.584 **	0.290 **	−0.286 **	0.767 **
	*p*			<0.001	0.691	0.292	0.916	<0.001	<0.001	<0.001	<0.001
Sorbitol	r				0.241 **	0.210 **	0.188 *	0.585 **	0.132	0.115	0.732 **
	*p*				0.002	0.009	0.019	<0.001	0.102	0.155	<0.001
Glucose	r					−0.572 **	0.112	−0.130	−0.216 **	0.073	0.218 **
	*p*					<0.001	0.164	0.107	0.007	0.364	0.006
Sucrose	r						0.241 **	0.778 **	0.086	−0.206 *	0.352 **
	*p*						0.003	<0.001	0.289	0.010	<0.001
Total acid	r							0.287 **	−0.866 **	−0.679 **	0.236 **
	*p*							<0.001	<0.001	<0.001	0.003
Total sugar	r								0.149	−0.273 **	0.832 **
	*p*								0.064	0.001	<0.001
Sugar/acid	r									0.587 **	0.166 *
	*p*									<0.001	0.039
pH	r										−0.216 **
	*p*										0.007

*: *p* ≤ 0.05; **: *p* ≤ 0.01.

## Data Availability

Not applicable.

## Supplementary Materials

The following are available online at https://www.mdpi.com/article/10.3390/foods10122950/s1, Table S1: The Sugars, acids, sugar/acid ratio, soluble solids, and pH of fruits of apple cultivars grown in Yuncheng, Shanxi Province, Table S2:The Sugars, acids, sugar/acid ratio, soluble solids, and pH of fruits of apple cultivars grown in Linfen, Shanxi Province, Table S3: The Sugars, acids, sugar/acid ratio, soluble solids, and pH of fruits of apple cultivars grown in Jinzhong, Shanxi Province, Figure S1:PCA model of cultivated apples by ripening time: (A) Scores plot; (B) Loadings plot. Aug represents August, Sep represents September and Oct represents October.
