# Supplementary material for: Effects of Genetic Background and Altitude on Sugars, Malic Acid and Ascorbic Acid in Fruits of Wild and Cultivated Apples (Malus sp.)
Source: Foods. 2021 Nov 30;10(12):2950. doi: 10.3390/foods10122950 (PMC8701241; doi:10.3390/foods10122950)
Supplement: Supplementary file 1 [file foods-10-02950-s001.zip › foods-1446457-supplementary.pdf]

Supplementary Materials

Table S1. The Sugars, acids, sugar/acid ratio, soluble solids, and pH of fruits of apple cultivars grown in Yuncheng, Shanxi Province.

Cultivars	Malic acid (mg/g)	Ascorbic acid (mg/g)	Fructose (mg/g)	Sorbitol (mg/g)	Glucose (mg/g)	Sucrose (mg/g)	Total acid (mg/g)	Total sugar (mg/g)	Sugar/acid	pH	Soluble solids (°Brix)
Gala	2.11±0.14e	0.08±0.02e	66.58±1.55a	2.78±0.45cd	21.40±0.62e	41.15±4.40a	2.19±0.15f	129.92±5.63b	59.43±2.44a	4.35±0.08bc	13.14±0.26cd
Xinshiji	4.39±0.57a	0.25±0.05bcd	62.97±3.64bc	8.70±2.08a	36.43±1.92a	22.51±7.78ef	4.64±0.56a	127.44±8.80b	27.63±1.87f	4.20±0.10ef	14.88±0.75b
Starkrimson	3.75±0.29b	0.24±0.23bc	53.17±3.27fg	3.30±0.56cd	23.49±2.46de	31.41±4.85bcd	3.99±0.34b	111.37±5.70ef	28.07±2.25f	4.33±0.05c	12.00±0.35e
Golden Delicious	2.49±0.30d	0.1±0.04de	52.75±4.27g	1.60±0.27e	22.31±1.22e	38.83±6.64ab	2.59±0.33e	113.36±4.65de	44.32±5.48bc	4.41±0.06b	11.78±0.11e
Red General Fuji	3.21±0.21c	0.18±0.14cde	56.70±2.10ef	2.81±0.41cd	25.00±1.23d	26.18±4.93de	3.39±0.23cd	107.74±7.05ef	31.97±4.03e	4.17±0.02f	11.58±0.38e
Nagafu No.2	3.26±0.23c	0.33±0.08ab	61.11±1.06c	5.77±1.04b	32.30±1.83b	31.92±3.62bcd	3.60±0.25c	131.10±2.66b	36.56±2.07de	4.31±0.02cd	13.46±0.59c
Huimin Fuji	3.03±0.17c	0.19±0.07cde	55.46±3.93efg	3.78±0.32c	28.49±3.11c	35.53±7.46abc	3.22±0.20d	120.93±5.68c	37.67±2.81d	4.30±0.02cd	12.80±0.34d
Yantai Fuji No.3	2.39±0.07de	0.09±0.04e	57.44±0.75de	3.27±0.13cd	30.82±0.56b	28.46±2.82cde	2.47±0.11ef	119.98±3.74cd	48.53±1.22b	4.24±0.02de	12.82±0.08d
Yantai Fuji No.6	2.37±0.08de	0.07±0.02e	60.27±0.70cd	2.38±0.04de	27.57±0.24c	15.81±3.02f	2.44±0.10ef	106.03±2.78f	43.42±0.75c	4.29±0.01cd	11.76±0.09e
Qinguan	2.70±0.08d	0.45±0.10a	65.78±1.59ab	8.83±0.51a	35.50±0.67a	41.01±7.28a	3.15±0.17d	149.12±5.97a	47.47±4.34b	4.48±0.03a	15.86±0.13a

Table S2. The Sugars, acids, sugar/acid ratio, soluble solids, and pH of fruits of apple cultivars grown in Linfen, Shanxi Province.

Cultivars	Malic acid (mg/g)	Ascorbic acid (mg/g)	Fructose (mg/g)	Sorbitol (mg/g)	Glucose (mg/g)	Sucrose (mg/g)	Total acid (mg/g)	Total sugar (mg/g)	Sugar/acid	pH	Soluble solids (°Brix)
Zhongqiuwang	3.22±0.33c	0.37±0.04a	69.43±2.00a	2.99±0.38cd	20.56±2.16de	34.61±12.73de	3.59±0.37d	127.59±13.23cd	35.73±4.15b	4.03±0.07d	13.74±0.81c
Starkrimson	2.61±0.35d	0.09±0.03c	54.98±2.79cd	3.31±0.87cd	21.68±1.25cde	46.63±8.84bc	2.71±0.35e	126.59±10.91cd	47.31±6.29a	4.24±0.05ab	12.06±0.70de
Golden Delicious	5.05±0.27a	0.28±0.11ab	54.06±3.07cd	3.54±0.52cd	22.49±2.39bcd	40.58±5.57cd	5.32±0.36a	118.65±5.91de	22.39±2.17c	4.01±0.05d	12.70±0.47d
Red General Fuji	4.23±0.30b	0.30±0.19ab	69.12±2.98a	5.30±0.80b	19.01±1.81e	53.53±3.62ab	4.53±0.16b	145.77±4.64b	32.17±0.71b	4.00±0.06d	14.00±0.34bc
Nagafu No.2	4.25±0.48b	0.25±0.05ab	64.37±3.46b	7.53±1.07a	23.64±2.52bc	63.04±7.18a	4.50±0.51b	158.00±6.64a	35.36±3.27b	4.04±0.09d	15.06±0.47a
Huimin Fuji	3.34±0.32c	0.18±0.09bc	57.44±3.58c	2.87±0.74d	23.28±1.97bcd	27.25±9.40e	3.52±0.37d	110.84±11.07e	31.75±4.70b	4.16±0.06bc	11.60±1.08e
Yantai Fuji No.3	3.59±0.47c	0.37±0.09a	65.34±2.49b	3.99±0.47c	29.11±2.15a	37.65±6.69cd	3.96±0.53cd	136.08±5.68c	34.86±5.04b	4.07±0.06d	14.76±0.34ab
Qiufu No.1	4.07±0.31b	0.16±0.07bc	70.81±3.66a	6.61±1.03a	25.24±1.31b	44.90±3.86bcd	4.24±0.30bc	147.57±4.83b	34.93±2.12b	4.09±0.03cd	15.48±0.52a
Qinguan	3.30±0.25c	0.26±0.19ab	51.84±0.61d	5.47±0.85b	23.39±2.34bcd	43.43±6.82bcd	3.56±0.41d	122.29±6.08d	34.84±4.91b	4.30±0.08a	12.34±0.15de

Supplementary Materials

Table S3. The Sugars, acids, sugar/acid ratio, soluble solids, and pH of fruits of apple cultivars grown in Jinzhong, Shanxi Province.

Cultivars	Malic acid (mg/g)	Ascorbic acid (mg/g)	Fructose (mg/g)	Sorbitol (mg/g)	Glucose (mg/g)	Sucrose (mg/g)	Total acid (mg/g)	Total sugar (mg/g)	Sugar/acid	pH	Soluble solids (°Brix)
Gala	2.81±0.38d	0.10±0.06cd	64.85±5.33b	2.98±0.74f	18.05±2.09c	48.47±2.74ab	2.90±0.41d	132.85±9.75cd	46.16±4.04d	4.17±0.07d	13.36±0.99c
Xiali	2.12±0.25efg	0.11±0.02cd	74.23±5.12a	5.77±0.71c	22.37±2.90a	43.19±3.57b	2.23±0.26ef	145.56±8.31b	65.73±5.21a	4.18±0.03d	15.60±1.01a
American No.8	2.39±0.33ef	0.13±0.03bc	58.81±2.97c	4.66±0.93cde	10.39±0.82d	53.91±6.60a	2.52±0.35def	127.77±8.94d	51.46±7.97cd	4.30±0.07c	12.44±0.60d
Liuyuehong	1.95±0.03g	0.20±0.04ab	63.99±0.85b	10.52±0.25a	18.04±0.30c	53.48±1.63a	2.15±0.05ef	146.03±2.83b	68.04±0.96a	4.55±0.01a	14.64±0.11b
Lihong	2.49±0.02de	0.11±0.05cd	65.68±0.61b	4.79±0.26cde	18.77±0.09c	54.12±0.37a	2.6±0.06de	143.35±1.04bc	55.14±1.52bc	4.22±0.01d	13.98±0.04bc
Starking Delicious	2.10±0.14efg	0.05±0.03d	67.52±2.24b	4.01±0.51def	20.18±1.91abc	34.05±2.92c	2.14±0.12ef	125.76±4.26d	58.92±5.34b	4.44±0.05b	13.30±0.31c
Starkrimson	2.05±0.06fg	0.05±0.02d	65.10±4.20b	5.03±1.17cd	21.89±1.78ab	33.11±3.49c	2.10±0.08f	125.13±5.68d	59.73±4.44b	4.57±0.04a	13.62±0.72c
Golden Delicious	4.52±0.20b	0.26±0.12a	66.91±2.85b	4.31±0.78de	19.65±1.66bc	53.79±8.36a	4.78±0.21b	143.50±10.02b	30.10±2.68f	4.08±0.04e	14.14±0.63bc
Red General Fuji	3.68±0.42c	0.20±0.04ab	65.50±2.63b	3.64±1.11ef	20.33±0.84abc	52.37±4.5a	3.88±0.44c	140.58±7.40bc	36.49±3.08e	4.06±0.07ef	13.40±0.54c
Nagafu No.2	5.01±0.60a	0.28±0.09a	74.04±2.02a	7.68±1.23b	21.42±2.09ab	55.71±9.48a	5.29±0.67a	157.81±10.23a	30.32±5.10f	4.01±0.02f	15.48±0.75a

Supplementary Materials

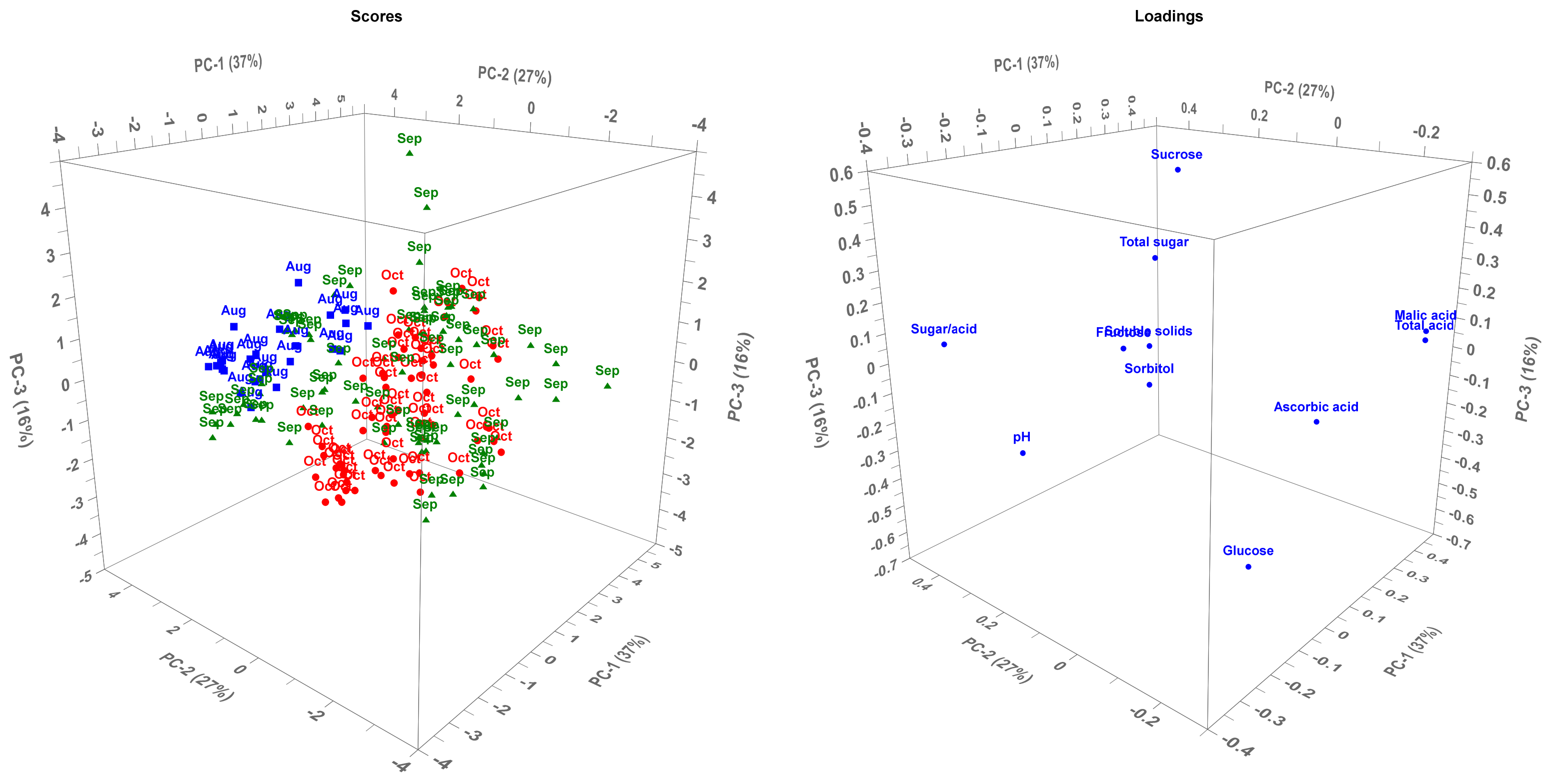

(**a**) (**b**)

Figure S1. PCA model of cultivated apples by ripening time: (a) Scores plot; (b) Loadings plot. Aug represents August, Sep represents September and Oct represents October.
